# Redefining mouse transgenesis with CRISPR/Cas9 genome editing technology

**DOI:** 10.1186/s13059-018-1409-1

**Published:** 2018-02-28

**Authors:** Gaetan Burgio

**Affiliations:** Department of Immunology and Infectious Disease, The John Curtin School of Medical Research, The Australian National University, Canberra, Australia

## Abstract

The generation of genetically modified alleles in mice using conventional transgenesis technologies is a long and inefficient process. A new study shows that the in situ delivery of CRISPR/Cas9 reagents into pregnant mice results in a high efficiency of editing, and enables the rapid generation of both simple and complex alleles.

To determine how a gene functions, interacts with other genes, or how its dysregulation or absence influences disease, the generation of modified alleles in model organisms, including mice, rats, *Drosophila*, zebrafish or *Caenorhabditis elegans*, is an incredibly powerful tool. Among these model organisms, the laboratory mouse plays a distinct role in biomedical research because of its genomic proximity with the human genome, its similar physiology to humans, and its ability to be genetically manipulated. However, the generation of these mutant mice requires a fastidious and demanding process that relies on a highly skilled team and expensive equipment that is only available in specialized facilities. The process of creating a knockout or knockin mouse allele was established over 30 years ago by Smithies, Evans and Capecchi [1]. Their method necessitates the culture and genetic modification of mouse embryonic stem cells by homologous recombination, with selection cassettes replacing a critical exon for a knockout allele, or two loxP sites flanking a critical exon in addition to the selection cassettes for a knockin allele, being a routine strategy for allelic replacement. The genetically modified embryonic stem cells are then drug selected and microinjected into mouse blastocysts. The microinjected blastocysts are finally implanted into pseudopregnant females by surgical transfer (Fig. 1a). The time frame to generate these mice is long, on average 1–2 years, and the efficiency is relatively low due to the complexity of this procedure. Recent studies, including two papers published recently in *Genome Biology* [2, 3], have used CRISPR/Cas9 genome editing technology to improve and simplify this procedure.

**Fig. 1:**
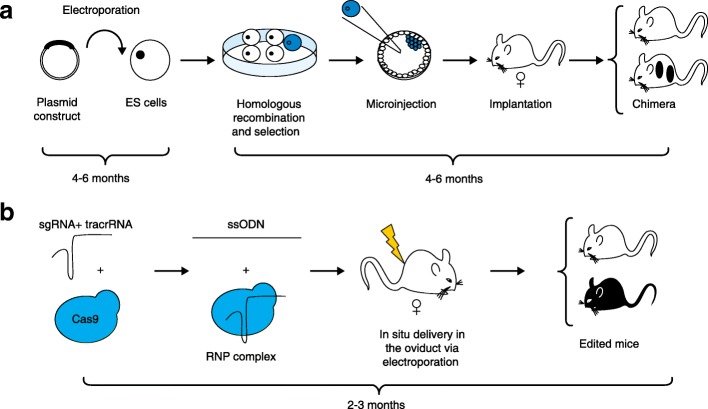
**a** Generation of knockout and knockin alleles using embryonic stem (*ES*) cell technology in mice. A cloning procedure is undertaken to insert the construct into a plasmid vector as a template to replace the endogenous locus. This template could be a drug-selection cassette only (knockout) or an exon flanked with two loxP sites, or a more complex feature (knockin). These vectors contain a positive and negative selection cassette. The plasmid is then electroporated into the ES cells and then drug selected in vitro. After verification that the sequence is correctly inserted, the cells are microinjected into a blastocyst, before being surgically transferred into pseudopregnant females. The chimeric progenies will be genotyped to ensure the expected construct is correctly inserted into the genome by homologous recombination. **b** Generation of complex alleles using improved-genome editing via oviductal nucleic acid delivery (i-GONAD) technology. One or two single guide RNAs (*sgRNA*) are designed to either disrupt a critical exon (knockout) or remove an entire exon for replacement with a repair template (knockin). The sgRNAs are synthesized, or in vitro transcribed, and then complexed with the tracrRNA and then Cas9 protein to form a ribonucleoprotein (*RNP*) complex. The RNPs are in situ electroporated with a long single-stranded oligonucleotide repair template (*ssODN*) into the oviduct of a pregnant female. The progenies are genotyped to ascertain successful editing of the gene of interest

## How CRISPR/Cas9 has transformed mouse transgenesis technologies

The emergence of CRISPR/Cas9 gene editing technology with its simplicity, versatility, and efficiency has considerably improved the time frame and the process of creating these modified alleles. The technology has enabled rapid production of knockout, conditional alleles or mice carrying single point mutations, which mimic those in human patients, in only several weeks. For the generation of knockout alleles, the microinjection of a single guide RNA (sgRNA) in zygotes is sufficient to create indels in a critical exon, inducing a frameshift mutation and therefore functionally abolishing the gene of interest [4]. However, the generation of more complex alleles, including conditionals, is more challenging and requires at least two sgRNAs and a repair template in the form of two short single-stranded DNA (ssDNA) repair templates each containing a loxP site [5]. The repair template replaces the endogenous locus of interest by homology-directed repair after Cas9 cleavage and the double-strand break (DSB) of the region of interest. Unfortunately, the process of generating these conditional alleles using programmable nucleases remains lengthy and relatively inefficient, as it requires microinjection of the DNA and the correct insertion of multiple repair templates into the same allele of the mouse genome, without recombination or mutation within the repair template or the exon of interest.

## Rapid and efficient generation of conditional alleles using Easi-CRISPR

A first research article from Quadros and colleagues aimed to improve the generation of conditional alleles in mice using programmable nucleases [2]. The authors made the simple observation that since the efficiency to repair DNA after DSB is higher for the homology-directed repair pathway than homologous recombination, the delivery of a longer repair template would result in a higher efficiency for generating mutant alleles. This technique, called efficient addition with ssDNA inserts-CRISPR (Easi-CRISPR), involves targeting by two sgRNAs which flank the endogenous exon and are complexed with Cas9 to form a ribonucleoprotein complex for cellular delivery. The exon is replaced after the DSB of the DNA and repaired with a long-stranded oligonucleotide template containing two loxP sites and spanning the entire exon. The authors demonstrated the power of this approach by showing a high efficiency in editing and allelic replacement, averaging a 50% success rate, and up to 100% editing for certain alleles, which is a marked improvement compared with conventional methods. Future work and replication studies from various research groups and transgenesis core facilities will confirm or disprove these observations. However, while efficient, this technique does not solve the limiting issues of CRISPR/Cas9, such as the requirement for highly trained staff and the use of an expensive microinjection apparatus only available in transgenesis core facilities.

## Combining CRISPR/Cas9 gene editing technology with an in situ delivery of the reagents in the oviduct: i-GONAD

A second research paper considerably simplified the procedure of generating complex alleles using CRISPR/Cas9 technology [3]. Ohtsuka and colleagues reasoned that direct delivery of the CRISPR/Cas9 reagents into the mouse oviduct would be as efficient, and more effective, than microinjection and surgical transfer of zygotes [3]. This technique would bypass all lengthy and complex procedures, from the isolation of zygotes from the oviduct, to mouse embryo transfer into recipient females. The other advantage of this method would be the reduction of the number of animals required to generate a knockout or knockin allele, since it is no longer necessary to sacrifice females for zygote collection, and the authors show that the recipient females are able to be impregnated again after their first transgenic litter.

Ohtsuka and colleagues [3] postulated that in situ delivery of the CRISPR/Cas9 reagents to the mouse oviduct by electroporation would allow Cas9 protein access to the zygote DNA to edit the genome. To ascertain this hypothesis, Ohtsuka and colleagues optimized the delivery protocol of CRISPR/Cas9 reagents to the oviduct and determined the optimum editing efficiency was at 0.7 dpc. The authors then hypothesized that an in vivo delivery of the CRISPR/Cas9 reagents combined with an Easi-CRISPR approach would display a similar, if not better, efficiency of editing simple or complex alleles when compared with microinjection into mouse zygotes. They tested this approach, called improved-genome editing via oviductal nucleic acid delivery (i-GONAD) (Fig. 1b), and after optimization the success of editing observed was up to 97% for straight knockout alleles and 50% for gene tags, which gives similar results to microinjected zygotes [3]. Importantly, Ohtsuka and colleagues demonstrated the feasibility of this approach targeting various genes in multiple mouse strains. Interestingly, the recipient females could be used for multiple experiments, suggesting that it is possible to considerably reduce the number of mice needed to generate these modified alleles. Whereas the efficiency of the i-GONAD approach to generate complex alleles using CRISPR/Cas9 genome editing technologies seems remarkable and promising, the frequency of mosaicism appears to remain up to 30% of edited alleles. Future optimization of this technique, combined with replication studies from various research groups, will enable improvements to the technology, address technical hurdles, and hopefully enable the successful and efficient generation of conditional alleles using the i-GONAD technique.

## Conclusion and perspectives: what will be the future of mouse transgenesis?

CRISPR/Cas9 gene editing technology has considerably changed transgenesis technologies. In the last 3–5 years, at a rapid pace, remarkable achievements have been observed. Easi-CRISPR and i-GONAD techniques have the potential to entirely reshape the traditional route of generating modified alleles in mice if the techniques are widely adopted by many research groups and transgenesis core facilities. It is predictable that soon all conventional steps to efficiently generate knockout or knockin alleles in mice will be bypassed and the CRISPR/Cas9 reagents will be delivered in situ into the oviduct. It will require less highly skilled personnel or specialized equipment since a stereomicroscope and an electroporation device would suffice to generate editing for simple or complex alleles. Importantly, it will enable the reduction of animal usage inline with the 3Rs rule for animal work. I would predict the recent technological development in gene editing and assisted reproduction will redefine decades of transgenesis. The future will tell what the pace of these changes will be.

## Abbreviations

DSB: Double-strand break
Easi-CRISPR: Efficient addition with ssDNA inserts-CRISPR
i-GONAD: Improved-genome editing via oviductal nucleic acid delivery
sgRNA: Single guide RNA
ssDNA: Single-stranded DNA
