# Correction to: Redefining mouse transgenesis with CRISPR/Cas9 genome editing technology

**DOI:** 10.1186/s13059-018-1424-2

**Published:** 2018-03-26

**Authors:** Gaetan Burgio

**Affiliations:** Department of Immunology and Infectious Disease, The John Curtin School of Medical Research, The Australian National University, Canberra, Australia

## Correction

In the recent Research Highlight [1], it has been highlighted that part b of Fig. 1 was incorrectly labelled as “sgRNA + tracrRNA” instead of “sgRNA (crRNA + tracrRNA)”. An updated Fig. 1, including also the amended figure legend has therefore been provided below.

**Fig. 1:**
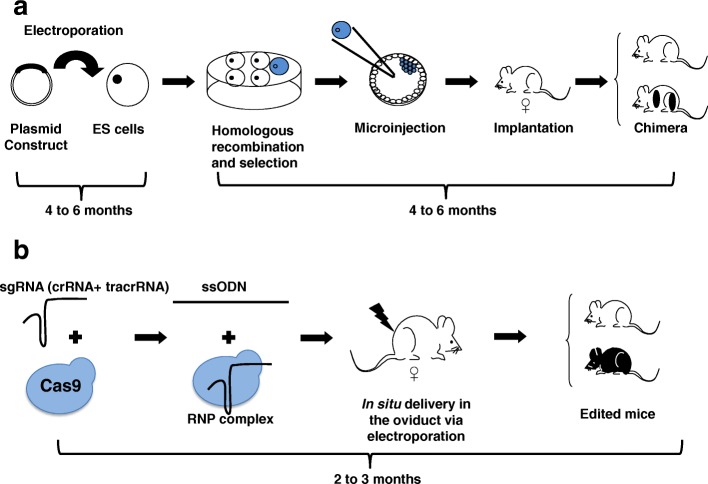
**a** Generation of knockout and knockin alleles using embryonic stem (ES) cell technology in mice. A cloning procedure is undertaken to insert the construct into a plasmid vector as a template to replace the endogenous locus. This template could be a drug-selection cassette only (knockout) or an exon flanked with two loxP sites, or a more complex feature (knockin). These vectors contain a positive and negative selection cassette. The plasmid is then electroporated into the ES cells and then drug selected in vitro. After verification that the sequence is correctly inserted, the cells are microinjected into a blastocyst, before being surgically transferred into pseudopregnant females. The chimeric progenies will be genotyped to ensure the expected construct is correctly inserted into the genome by homologous recombination. **b** Generation of complex alleles using improved-genome editing via oviductal nucleic acid delivery (i-GONAD) technology. One or two single guide RNAs (sgRNA) are designed to either disrupt a critical exon (knockout) or remove an entire exon for replacement with a repair template (knockin). The sgRNAs (crRNA/tracRNA) are synthesized, or in vitro transcribed, and then complexed with the tracrRNA and then Cas9 protein to form a ribonucleoprotein (RNP) complex. The RNPs are in situ electroporated with a long single-stranded oligonucleotide repair template (ssODN) into the oviduct of a pregnant female. The progenies are genotyped to ascertain successful editing of the gene of interest

Additionally, in the section entitled, “Rapid and efficient generation of conditional alleles using Easi-CRISPR”, there is a sentence currently written:

“This technique, called efficient addition with ssDNA inserts-CRISPR (Easi-CRISPR), involves targeting by two sgRNAs which flank the endogenous exon and are complexed with Cas9 to form a ribonucleoprotein complex for cellular delivery”.

This is incorrect and should read:

This technique, called efficient addition with ssDNA inserts-CRISPR (Easi-CRISPR), involves targeting by two synthetic sgRNAs (crRNA/tracRNA) which flank the endogenous exon and are complexed with Cas9 to form a ribonucleoprotein complex for cellular delivery.

I apologise for these errors which have been acknowledged and corrected in this Correction article.
